# Species distribution models advance our knowledge of the Neanderthals’ paleoecology on the Iranian Plateau

**DOI:** 10.1038/s41598-020-71166-9

**Published:** 2020-08-28

**Authors:** Masoud Yousefi, Saman Heydari-Guran, Anooshe Kafash, Elham Ghasidian

**Affiliations:** 1 Department of Environmental Sciences, Faculty of Natural Resources, University of Tehran, Karaj, Iran; 2 Stiftung Neanderthal Museum, Mettmann, Germany; 3 DiyarMehr Institute for Palaeolithic Research, Kermanshah, Iran

**Keywords:** Ecology, Environmental sciences

## Abstract

Neanderthals (*Homo neanderthalensis*) were distributed across a vast region from Europe to western and Central Asia. The Neanderthals’ paleoecology and distribution has been extensively studied in Europe where the species originated. However, very little is known about their paleoecology in south-western Asia. Here, we employed species distribution modelling and 45 Middle Palaeolithic (c. 200,000–40,000 years BCE) sites location associated with fossil and/or lithic artefacts made by the Neanderthals to examine the expansion of the Neanderthals on the Iranian Plateau in south-western Asia. We estimated the niche overlap between Neanderthals and wild goat, wild sheep and Persian gazelle by modelling their past distribution using 200, 143 and 110 occurrence records respectively. The results show that Neanderthals had highest niche overlap with wild goat in the study area. This analysis revealed that the most suitable Neanderthals’ habitats in south-western Asia were located in the Zagros Mountains stretches from north-western and western and some isolated patches in the central parts of the Iranian Plateau. The annual precipitation and maximum temperature of the warmest month were the most important predictor of the species’ distribution. This finding shows that the southern edge of the Neanderthals distribution was limited by warm summer. Our results provide important information for future field investigations and excavations in the area.

## Introduction

Our closest relative, Neanderthals, separated from the modern human lineage around 800,000–1,200,000 years ago^1^ and became extinct around 40 kya^2^. During that time, Neanderthals were distributed across Europe and Asia (ibid) and experienced several glacial and interglacial periods. Besides of the questions concerning their co-existence with *Homo sapiens* and replacement, there are still many other basic questions which are not fully answered such as Neanderthals’ expansions and their adaptation capabilities in different environments. Despite Neanderthals being extensively studied across Europe^3–10^ and in the Levant^11–13^, very little is known about its paleoecology on the Iranian Plateau. Knowing Neanderthals paleoecology promotes deciphering the drivers of Neanderthal range expansion, their extinction and identifying the *Homo neanderthalensis* and *Homo sapiens* contact zone and understanding our own species past ecology.

The Iranian Plateau, the southern east-most expansion of the Neanderthals, is located in south-western Asia and has been recognized as an important dispersal corridor for Pleistocene hominin species and played a prominent role in their evolution^14–16^. Over the past decades, Middle Palaeolithic (c. 200,000–40,000 years BCE) occupations have been discovered in a number of different Palaeolithic sites in the Iranian Plateau^15–19^, but no study was performed to determine the species paleo-distribution and identify abiotic drivers of its distribution, both of which are necessary in understanding the Middle Palaeolithic species paleoecology.

Recently, Species Distribution Models (SDMs) have found many applications in paleoecology and paleobiogeography^20,21^. Rapid advances in species distribution modelling^22,23^ and availability of environmental data characterizing past climatic conditions^24^ made it possible to reconstruct the past distribution of species^21^. This gives paleoecologists and historical biogeographers an opportunity to study and test hypotheses regarding species dispersal and evolution^20,25^. These models were recently applied in studying paleoecology of hominin species and increased our knowledge of their paleodistributions^26–29^. For example, Benito et al.^28^ reconstructed the ecological niche and distribution of the Neanderthals during the Last Interglacial in Europe and the Irano-Turanian region. They found that climate was the most important predictor of the species’ distribution at the continental scale, while topography shaped its distribution at the local scale.

Here, we aim to reconstruct the distribution of the Neanderthals on the Iranian Plateau and estimate their niche overlap with their main prey species; wild goat (*Capra aegagrus*), wild sheep (*Ovis orientalis* and *Ovis vignei*) and Persian gazelle (*Gazella subgutturosa*) using species distribution modelling, as these are the species that were the top diet among the Neanderthals in the key Middle Palaeolithic sites around and on the Iranian Plateau^15,30–33^. Wild goat was reported as the most frequent food resource in the Neanderthals diet in the Zagros Mountains^33^. Thus we expect higher niche overlap between Neanderthals and wild goat compared to wild sheep and Persian gazelle.

Previous studies have shown that distribution of species at the global and continental scales are shaped by climatic factors while topography determines species distribution at regional and local scales^34^. Here, at the regional scale, we hypothesize that topography is the most influential factor in the Neanderthals expansion^28,34^.

## Results

### Paleo-distribution model of Neanderthals

**Figure 1:**
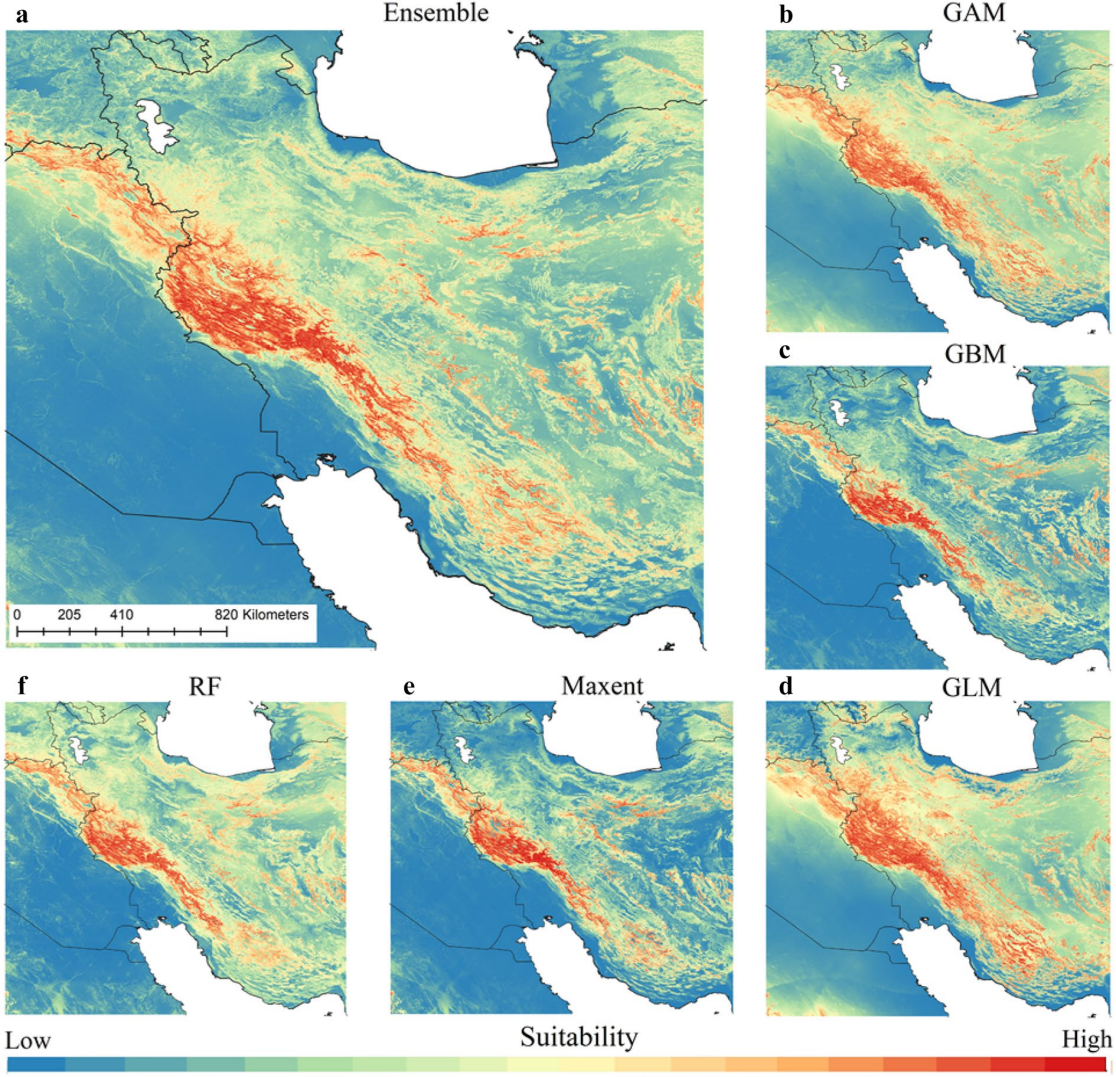
Paleo-distribution models of Neanderthals. Ensemble model (**a**) was created by combining Generalised Additive Model (**b**), Generalised Boosting Model (**c**), Generalized Linear Model (**d**), Maximum Entropy (**e**), Random Forest (**f**). The paleo-distribution models were mapped in QGIS 1.14.1 (https://qgis.org/en/site/).

Based on the ensemble model, Zagros Mountains and some patches in the central parts of the Iranian Plateau were the most suitable Neanderthals habitats. North of the Persian Gulf, Alburz Mountains and some isolated patches in the central Iran were found to have moderate suitability while northwest of Persian Gulf and the interior parts of the Mesopotamian lowlands (central and southern parts of modern Iraq) were identified with no suitability (Fig. 1). Our estimates showed that area of suitable habitat for the Neanderthals is 281,677 km^2^ across the study area. Results of assessing the predictive performance of models developed in this study showed that all models performed well in predicting the distribution of the species (Figs. 3a and S1–S3).

### Paleo-distribution models of prey species

**Figure 2:**
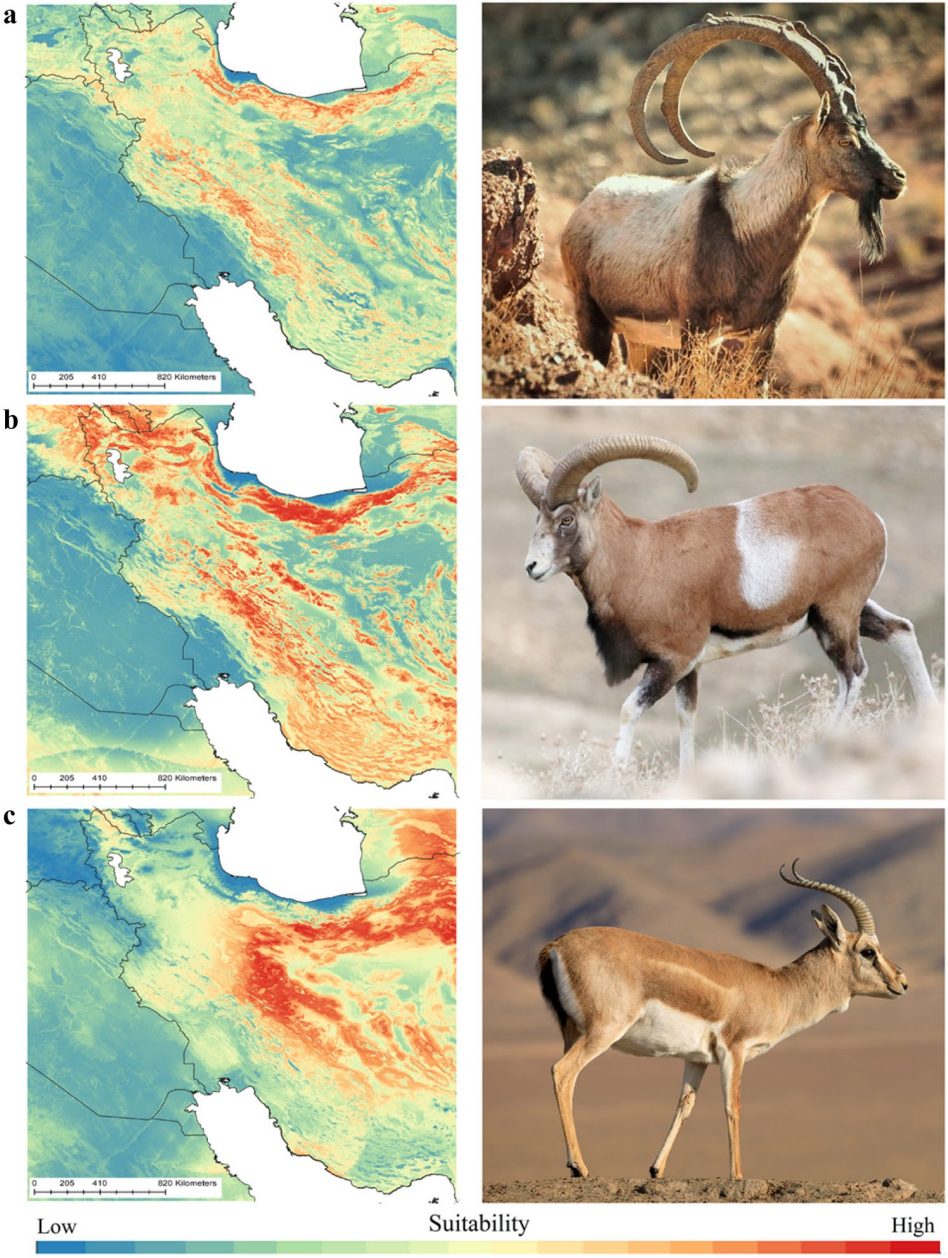
Paleo-distribution of the Neanderthals prey species*.* Past distribution models (ensemble distribution models) of wild goat (**a**), wild sheep (**b**) and Persian gazelle (**c**) (top left and low left photos by Mehdi Jalalpoor and middle left photo by Ali Saghafi). The paleo-distribution models were mapped in QGIS 1.14.1 (https://qgis.org/en/site/).

Results of hindcasting distribution of the three prey species showed that high altitude areas in the Zagros and Alborz Mountains and the mountains of central Iran were recognized with high habitat suitability for wild goat (Fig. 2a) and wild sheep (Fig. 2b) while low altitude areas in the central Iranian Plateau identified with high suitability for Persian gazelle (Fig. 2c).

### Variable importance and response curve

**Figure 3:**
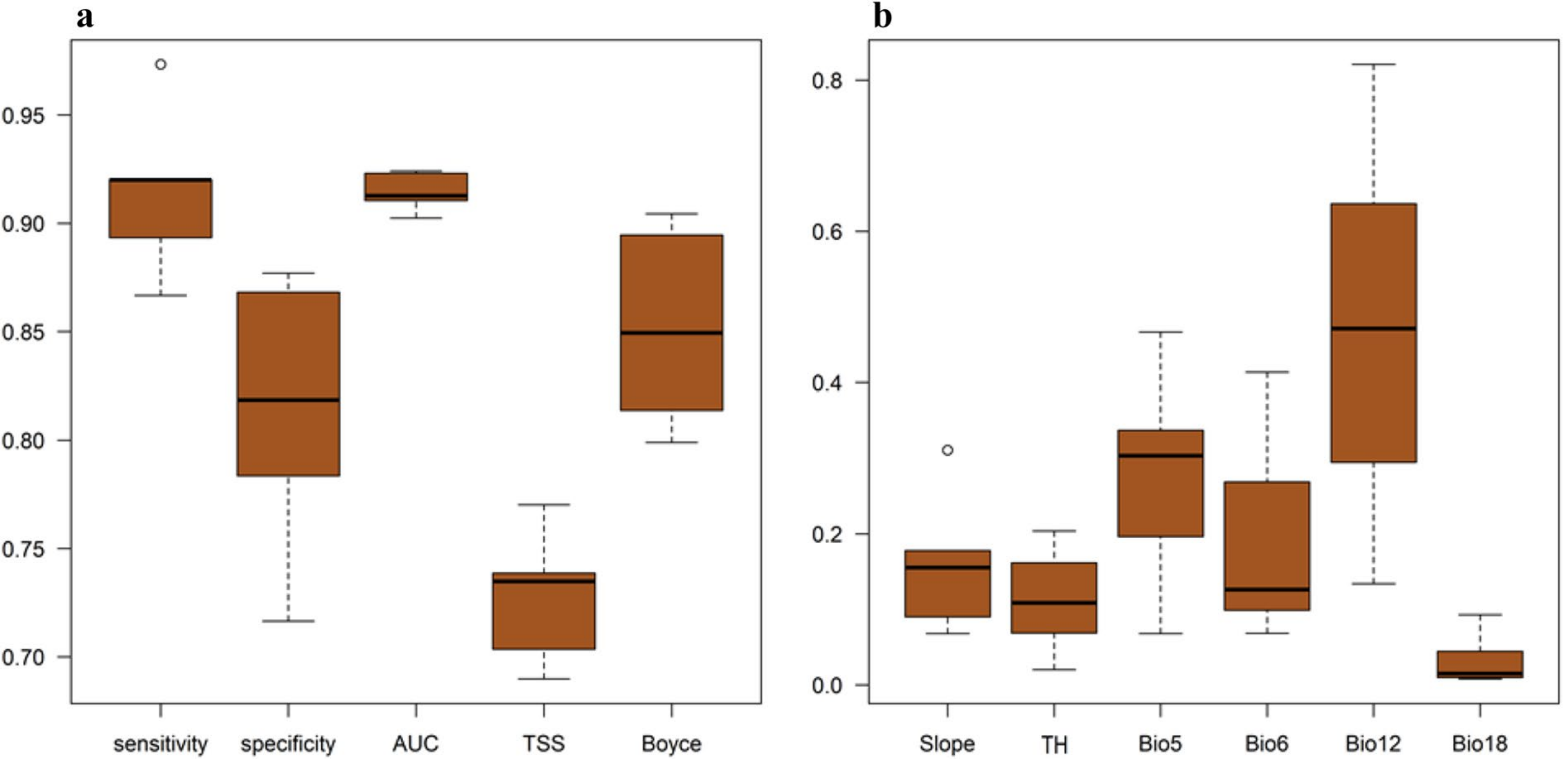
Results of model performance and variables importance. Results of model evaluation using sensitivity, specificity, AUC, TSS, and Boyce index (**a**) and variables importance test (**b**). Bio5: maximum temperature of the warmest month; Bio6: minimum temperature of the coldest month; Bio12: annual precipitation; Bio18: precipitation of the warmest quarter; TH: topographic heterogeneity. This figure created using the R software version 3.3.3 (https://www.r-project.org/).

**Figure 4:**
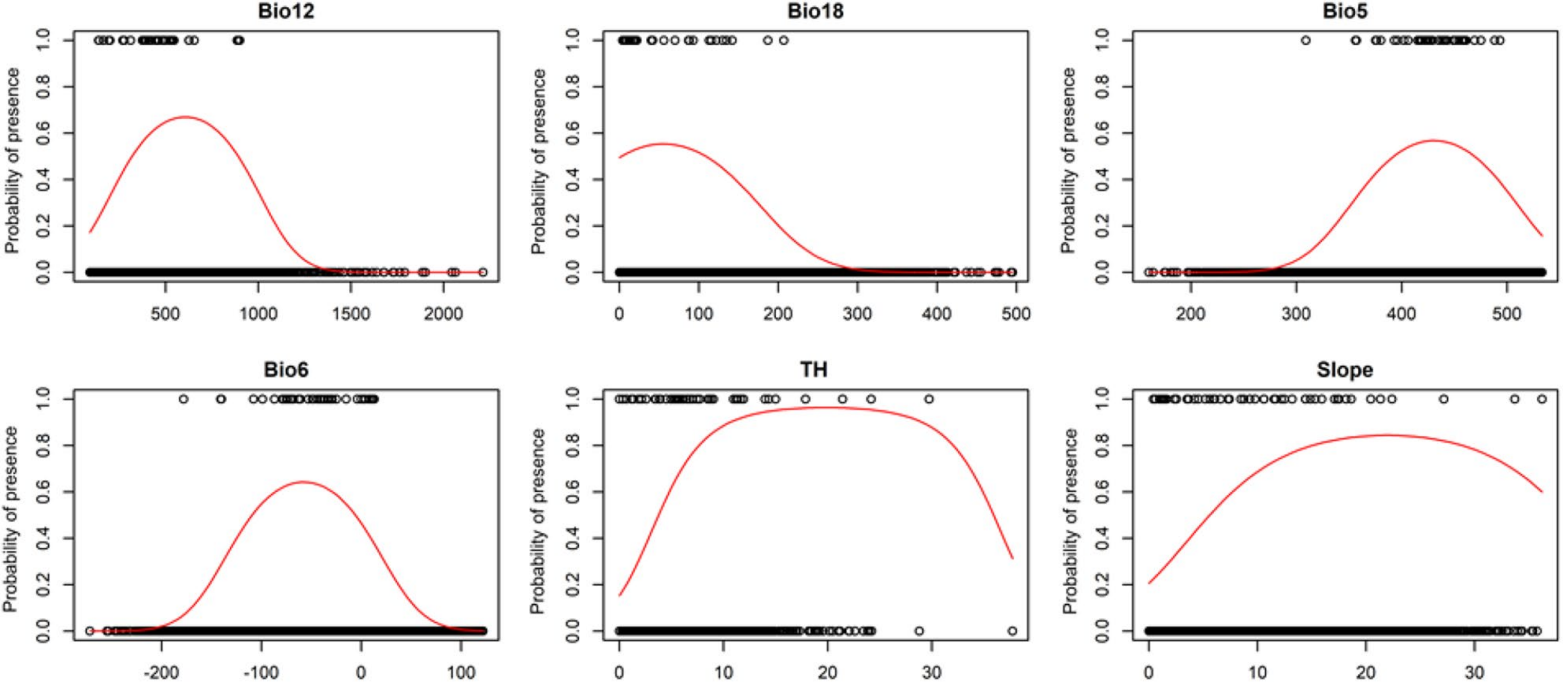
Neanderthals response to the environmental variables. Response curves showing how Neanderthals responded to the climatic and topographic variables. Bio5: maximum temperature of the warmest month; Bio6: minimum temperature of the coldest month; Bio12: annual precipitation; Bio18: precipitation of the warmest quarter; TH: topographic heterogeneity. This figure created using the R software version 3.3.3 (https://www.r-project.org/).

Our results showed that annual precipitation and maximum temperature of the warmest month were the most important predictor for the Neanderthal distribution (Fig. 3b). According to the response curves (Fig. 4), annual precipitation showed a normal distribution with an optimum around 600 mm. The maximum temperature of the warmest month showed a close-to-normal distribution with an optimum around 45 °C. Areas with moderate slopes and topographic heterogeneity yielded higher suitability for the species presence. Precipitation of the warmest quarter was identified as the most important determinant of wild goat and wild sheep distribution and slope as the most important predictor of Persian gazelle distribution (Figs. S1, S2 and S3).

### Niche overlap

Results of estimating niche overlap between Neanderthals and the three prey types showed that the Neanderthals and wild goat had the highest niche overlap (Schoener’s D = 0.77) followed by wild sheep (Schoener’s D = 0.72) and Persian gazelle (Schoener’s D = 0.66*).*

## Discussion

The Iranian Plateau was a vital migration route and dispersal corridor for Pleistocene hominin species^14^, however research on hominin paleoecology is limited in the plateau. In the present study we reconstructed the distribution of the Neanderthals in the Iranian Plateau for the first time and estimated their niche overlap with its main prey species. We found that the most suitable habitats of the Neanderthals were located in the west and northwest of the study area as well as some isolated patches in the central parts of the Iranian Plateau. We also found that climate shaped the species distribution at a regional scale.

Neanderthals niche overlap with its prey was not quantified across its distribution range before this study^28,35^ but species distribution models^22^ allowed us to estimate this archaic top human predator niche overlap with its major prey species. We found maximum niche overlap between Neanderthals and wild goat (Schoener’s D = 0.77) however, wild sheep also showed high niche overlap with the species (Schoener’s D = 0.72). This finding matches with our expectation that wild goat would have had the highest niche overlap with the species. Wild goat was the most frequently used food item by Neanderthals^30–33^. Wild goat and wild sheep are dominant herbivores in Zagros Mountains which were identified to have highest suitability for Neanderthals, but the distribution of Persian gazelle is different and most suitable habitats were located in central Iranian Plateau which characterized with lower annual precipitation and flat areas. Due to high niche overlap between Neanderthals and wild goat we recommend considering wild goat paleodistribution model in reconstructing ecological niche of Neanderthals.

Ecological niche modelling of the Neanderthals across Europe and the Irano-Turanian Region revealed that annual precipitation and minimum temperature of the coldest month were the most important predictor at the continental scale^28^. We found that at the regional scale, climatic factors are the most important predictors of the Neanderthals distribution, this is not in concordance with our hypothesis that states that topographic variables are more effective predictors of the Neanderthals distribution at the regional scale. Like for European Neanderthals, annual precipitation was the most important determinant of distribution of Iranian Neanderthals. Precipitation is important due to its effect on productivity of the habitats occupied by this species and its prey. The minimum temperature of the coldest month was the second most important predictor of European and Irano-Turanian populations with a sigmoidal trend while in Iranian populations the maximum temperature of the warmest month was the second most important variable with a normal distribution. This shows that the northern edge of the Neanderthals distribution was limited by cold winters^28^ while the southern edge by warm summer. Optimum temperature of the warmest month range was around 15 °C, from 30 °C in European and Irano-Turanian populations to 45 °C in Iranian populations.

However, we found that topography is not important at regional scale but it may be an important determinants of Neanderthals microhabitat use at local scale in Zagros Mountains^15^. According to the previous researches^15,19,36,37^ in the several areas of Khorramabad valley^35,36^ and Kermanshah^15,19^, a pattern of habitation choose by Neanderthal societies can be raised. Based on the Heydari-Guran and Ghasidian^19^ observation for Kermanshah, the Middle Palaeolithic occupations are desire mostly toward to the areas characterized by sharp contrast topography. Sharp contrast topography areas are a type of landform which includes flat plains immediately close to steep rugged mountains^19^. They argue this type of landform embraces a diverse ecological ecotone in a relatively small-scale area. It should be noticed that the above mentioned topographic condition matches well with the wild goat. According to Benito et al.^28^ topography was the most influential determinant of Neanderthals habitat suitability at the local scale. This highlighted the facts that different environmental variables influence species distribution at different spatial scales.

Only five confirmed records of Neanderthals fossils exist across the study area. The lack of Neanderthals fossils is due to limited field excavation and purposeful Palaeolithic research in the study area^15,19^. One other reason for the lack of fossils records is that Neanderthals coexisted with predators like hyenas^32,33^ so these animals probably exhumed shallow-buried Neanderthals in caves or rock shelters and stopped the fossilization process before it started^38^. SDMs are successfully used to find new populations of species and were suggested as a useful tool to identify potential areas for fieldwork and species monitoring^22^. Our model identified areas of suitable habitats in the study area which can be subject for future field examinations and excavations to discover new sites associated with the Neanderthals fossils.

## Conclusions

We reconstructed Neanderthals distribution on the Iranian Plateau, and estimated its niche overlap with its main prey species in the area. Our results revealed that Neanderthals distribution was determined by climatic variables at a regional scale. We documented maximum niche overlap between Neanderthals and wild goat among its prey species. This study showed how present day data can be used in studying paleoecology of an archaic human species which went extinct around 40,000 years ago. By applying present day distribution record of Neanderthals’ prey species we projected their past distribution to find which prey distribution better matches Neanderthals’ distribution. Nowadays, SDMs are frequently being used in archaeology and paleoanthropology^26,27^. SDMs allow archaeologists and paleoanthropologist to reconstruct distribution of an ancient culture and its interactions with environment across time and space^39,40^. Then it would be possible to identify drivers of historical cultures appearance and disappearance^39,40^. In addition, SDMs allow us to project species distribution in to the past to see where extant, or extinct species, were living in the past. Our research provides critical information for understanding Neanderthals paleoecology and environmental predictors of its distribution on the Iranian Plateau, an important geographic location for evolution and dispersal of Pleistocene hominin species.

## Materials and methods

### Neanderthals’ distribution data

**Figure 5:**
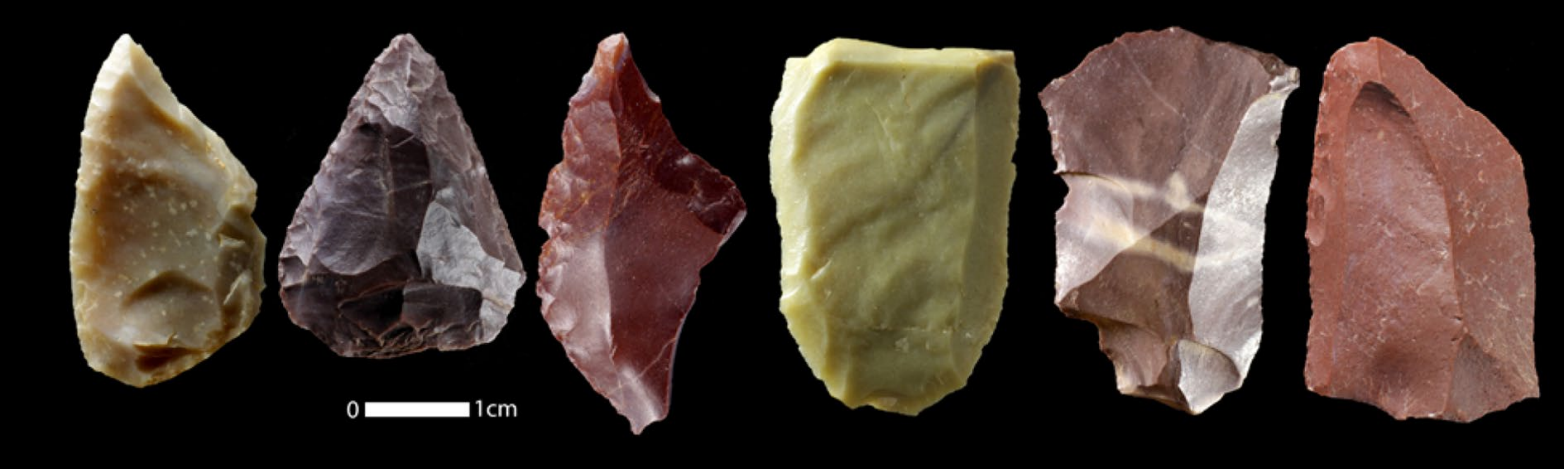
A collection of the lithic artefacts from Zagros Mountains, Kermanshah Region, Nawdarwan Valley. The lithic artefacts photographed by Saman Heydari-Guran.

**Figure 6:**
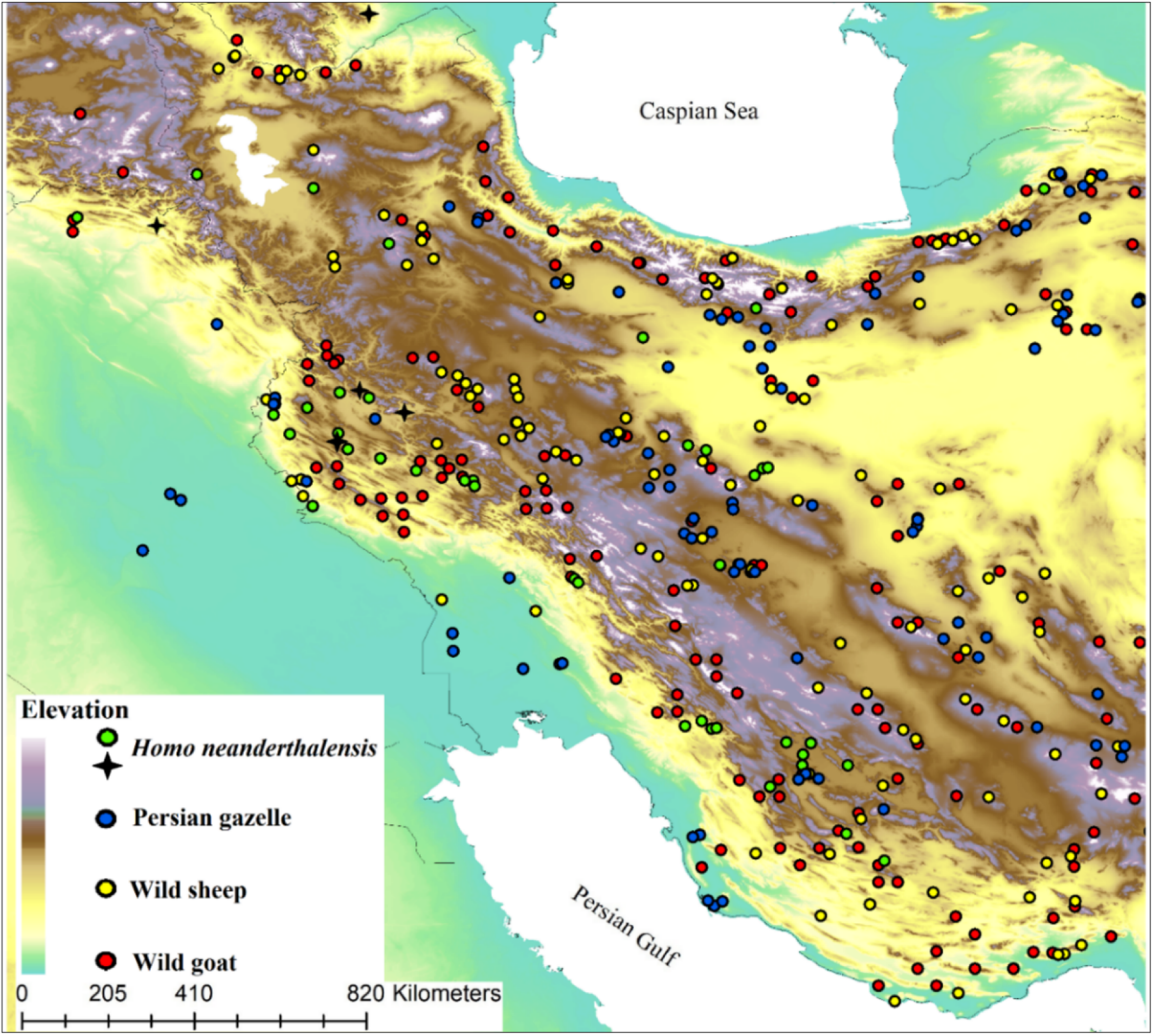
Presence records of Neanderthals and its prey species. Neanderthals physical remains are shown with black stare and sites associated with Middle Palaeolithic artefacts with green, wild goat with red, wild sheep with yellow and Persian gazelle with blue circles in a topographic overview of the study area. Elevation layer was obtained from the Shuttle Radar Topography Mission (SRTM) elevation model^57^ (https://srtm.csi.cgiar.org/).

Neanderthals distribution records were found from two sources; (1) Physical remains of the Neanderthal skeleton in Azokh in Armenia^41^, Shanidar in Iraqi-Kurdistan^42,43^, Wezmah Cave^44^, Bisetun Cave^45^, Bawa Yawan Rockshelter^18^, all located in Kermanshah region. (2) Surveyed and excavated sites associated with the Middle Palaeolithic assemblages including specific Mousterian artefacts^14,15,17,18,46,47^. We found 138 locations all associated with fossil and/or lithic artefacts (Fig. 5) made by the Neanderthals^18,19^. Some of these distribution records were close to each other and since species distribution modelling suffers from autocorrelation among distribution records we applied a filtering method to reduce autocorrelation among distribution records. Thus, we thinned our distribution records to 5 km meaning that we set a minimum distance of 5 km between all the occurrence points. This reduced our number of records to 45 (Fig. 6, Table S1).

### Prey data

*Homo neanderthalensis* is known as top-level carnivore which relied mostly on medium and large-sized herbivorous mammals^32,48^. We reviewed published books and papers which investigated materials obtained from the Neanderthals occupations to identify Neanderthals prey in the study area^15,31–33^. Neanderthals diet is highly diverse but here we chose three herbivorous mammals which were frequently hunted and used by the species based on published studies^15,33,46–51^; wild goat (*Capra aegagrus*), wild sheep (*Ovis gmelini* and *O. vignei*), and Persian gazelle (*Gazella subgutturosa*). For example, in a study by Perkins^49^ in the Shanidar Cave, 90 percent of the identified bones belong to the wild goat and wild sheep. These species are still the most important herbivorous mammals on the Iranian Plateau^52^. We combined distribution records of *O. gmelini* and *O. vignei* because the two species live in similar habitats in the study area. It should be noted that, the two were considered as single species, *O. orientalis*, by the International Union for Conservation of Nature until recently. We did not consider *G. bennettii* and *G. gazelle* since there is no evidence of presence of the two species in Zagros Mountains or being consumed by Neanderthals^15,33,46–51^. We obtained distribution records for the prey species from 12 years (2007–2018) of opportunistic observations across the study area and from published books, papers, atlases and theses^52–54^. We then carefully examined these distribution records, deleted duplicates and thinned to reduce autocorrelations^22^. In total, 200 records were obtained for wild goat, 143 for wild sheep and 110 for Persian gazelle (Fig. 5).

### Environmental data

To reconstruct Neanderthal distribution and quantify its ecological niches, we used topographic and past climatic variables which were identified as important drivers of its distribution^8,28^. Climatic variables (Bio5: maximum temperature of the warmest month; Bio6: minimum temperature of the coldest month; Bio12: annual rainfall; Bio18: precipitation of the warmest quarter) were obtained from Worldclim^24^. For topography we included slope and topographic heterogeneity in our model^28^. Slope was calculated using the terrain function in the Raster package^55^ in R environment^56^. The terrain function calculates slope as a function of elevation. We quantified topographic heterogeneity by measuring the standard deviation of elevation values in area grid cells of 1 km from a 90 m resolution. Elevation layer was obtained from the Shuttle Radar Topography Mission (SRTM) elevation model^57^. Collinearity among variables is an important source of bias in SDMs, so to make sure we did not include correlated variables in our models a variance inflation factor (VIF^58^) was calculated for the variables using the ‘vifstep’ function in the ‘usdm’ package^59^ and we found low collinearity among variables (VIF values: Bio5 = 4.032, Bio6 = 4.112, Bio12 = 1.574, Bio18 = 1.971, TH = 4.567, slope = 5.131).

### Reconstruction past distribution

Many algorithms exist to develop distribution models for a target species, but it is not well known which algorithms are the best for which purposes^23^. However, because different modelling methods can yield varying results, it is recommended to use an ensemble of several methods^60^. Ensemble approach allows us to simultaneously take into account results from multiple modelling approaches, thus producing more robust models^23,60^. In this study, in order to reconstruct Neanderthals distribution and hindcast the three prey species’ distribution, we used five algorithms: Generalized Linear Models (GLM^61^), Generalised Additive Models (GAM^62^), Generalised Boosting Models (GBM^63^), Maximum Entropy Modelling (Maxent^64^), and Random Forest (RF^65^). We then applied an ensemble approach^60^ to combine the predictions generated by different individual SDMs into a single final distribution model^60^. We calibrated the models using 80% of records drawn randomly and used as training data, and evaluated their performance using the remaining 20% of the data (test dataset). To show the relationship between habitat suitability and the values of each predictor within the study area we created response curves of all six climatic and topographic variables based on GLM model in R software version 3.3.3^56^.

### Assessing model performance

We evaluated the predictive performance of the models using the following metrics; area under the receiver operating characteristic curve (AUC)^66^, specificity, sensitivity^22^, the Boyce index^67,68^, the true skill statistic (TSS). AUC, specificity and sensitivity values range from 0 to 1, a value of 0.5 indicates that the performance of the model is not better than random, while values closer to 1.0 indicate better model performance^69^. Boyce index and TSS values range from − 1 to + 1, where + 1 indicates perfect performance and value of zero meaning random predictions^22^.

### Niche overlap analysis

Species distribution models allow us to estimate niche overlap between two or more competitors, predators and prey, and a single species niche trough time^22,25^. Here we calculated niche overlap between *H. neanderthalensis *as predator and three major prey of the species (wild goat, wild sheep and Persian gazelle). To end this, we used Schoener’s D niche overlap metric^70^ in ENMtools 1.4.4^25^. Schoener’s D ranges from 0 (no overlap; niches are completely different) to 1 (complete overlap; niches are identical).

## Supplementary information

Supplementary information

## Data Availability

All data needed to evaluate the conclusions in the paper are present in the paper and/or the Supplementary Materials, or the references cited here within.
