# Supplementary material for: Species distribution models advance our knowledge of the Neanderthals’ paleoecology on the Iranian Plateau
Source: Sci Rep. 2020 Aug 28;10:14248. doi: 10.1038/s41598-020-71166-9 (PMC7455561; doi:10.1038/s41598-020-71166-9)
Supplement: Supplementary file 1 — Supplementary information [file 41598_2020_71166_MOESM1_ESM.pdf]

Supplementary Information

Species distribution models advance our knowledge of the Neanderthals' paleoecology on the Iranian Plateau

Masoud Yousefi[1]*, Saman Heydari-Guran[2&3], Anooshe Kafash[1], Elham Ghasidian[2&3]

[1]Department of Environmental Sciences, Faculty of Natural Resources, University of Tehran, Karaj, Iran.

[2] Stiftung Neanderthal Museum, Germany.

[3] DiyarMehr Institute for Palaeolithic Research, Kermanshah, Iran.

*Corresponding author email address: yousefi52@ut.ac.ir

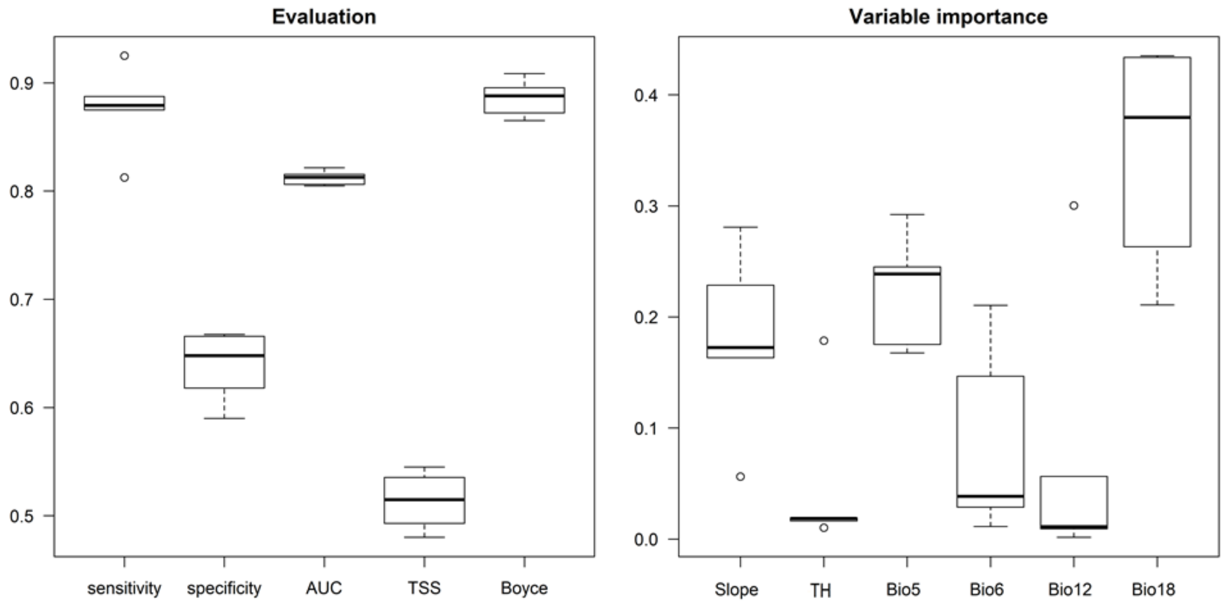

Figure S1. Results of model performance and variables importance of wild goat. Results of model evaluation using sensitivity, specificity, AUC, TSS, and Boyce index and variables importance test for wild goat. Bio5: maximum temperature of the warmest month; Bio6: minimum temperature of the coldest month; Bio12: annual precipitation; Bio18: precipitation of the warmest quarter; TH: topographic heterogeneity.

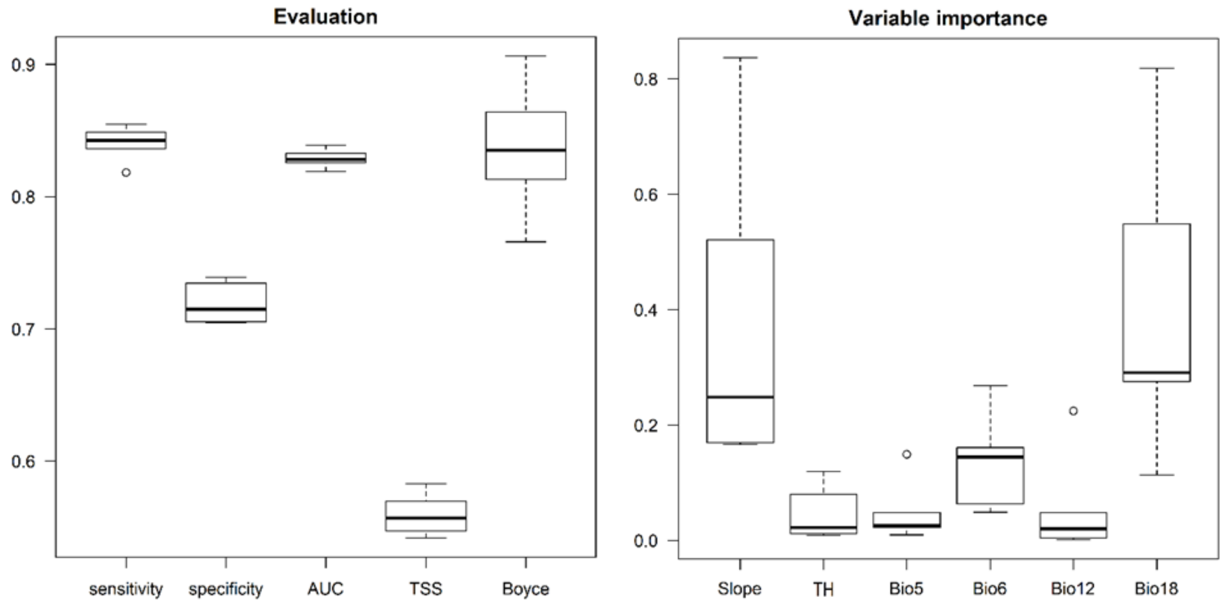

Figure S2. Results of model performance and variables importance of wild sheep. Results of model evaluation using sensitivity, specificity, AUC, TSS, and Boyce index and variables importance test for wild sheep. Bio5: maximum temperature of the warmest month; Bio6: minimum temperature of the coldest month; Bio12: annual precipitation; Bio18: precipitation of the warmest quarter; TH: topographic heterogeneity.

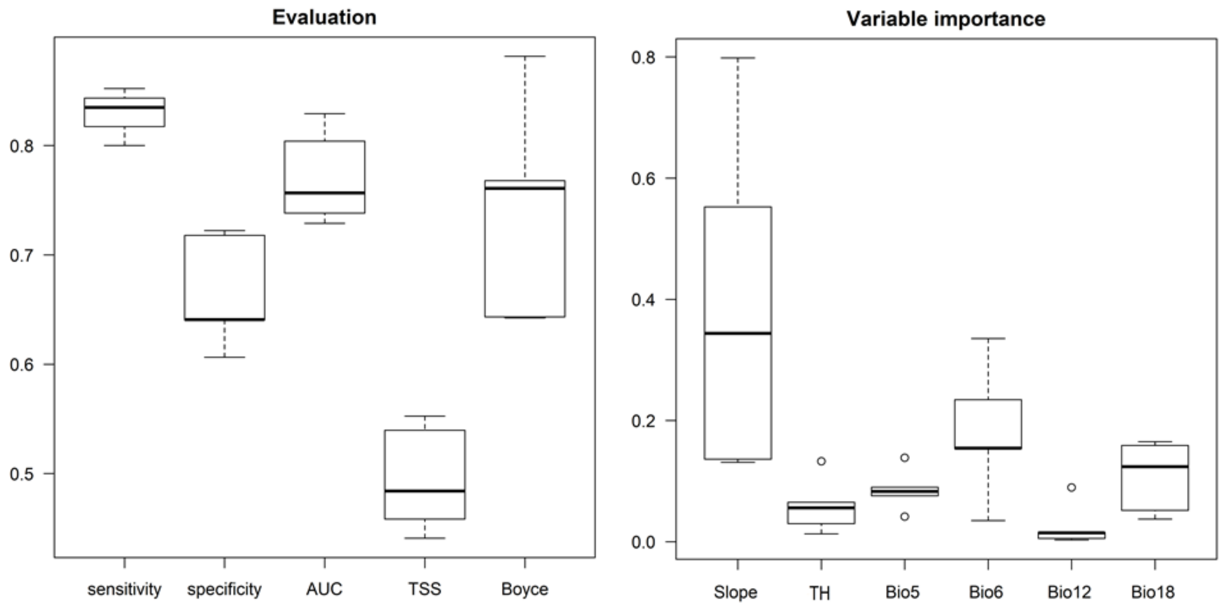

Figure S3. Results of model performance and variables importance of Persian gazelle**.** Results of model evaluation using sensitivity, specificity, AUC, TSS, and Boyce index and variables importance test for wild goat. Bio5: maximum temperature of the warmest month; Bio6: minimum temperature of the coldest month; Bio12: annual precipitation; Bio18: precipitation of the warmest quarter; TH: topographic heterogeneity.

Table S1. Information of 45 excavated sites associated with fossil and/or lithic artefacts made by the Neanderthals (Mousterian artefacts).

Number	Site	Latitude	Longitude	Province/Country	Artifacts	References
1	Azokh	39.61	46.98	Nagorno-Karabakh (Azerbaijan)	Neanderthal	King, T., Compton, T., Rosas, A., Andrews, P. Yepiskoposyan, L. & Asryan, L., Azokh Caves Hominin Remains. In: Fernández-Jalvo, Y., King, T., Yepiskoposyan, L., Andrews, P., (Eds.), Azokh Cave and the Transcaucasian Corridor (103–106). Dordrecht: Springer (2016).
2	Shanidar	36.83	44.22	Erbil, Kurdistan Region (Iraq)	Neanderthal	Solecki, R. Prehistory in Shanidar Valley, Northern Iraq. *Science* **139**, 179-193 (1963). Pomeroy, E., Mirazón Lahr, M., Crivellaro, F., Farr, L., Reynolds, T., Hunt, C.O. & Barker, G. Newly discovered Neanderthal remains from Shanidar Cave, Iraqi Kurdistan, and their attribution to Shanidar 5. *Journal of Human Evolution* **111**: 102–18 (2017).
3	Ishkaft Babkhal	36.70	44.47	Erbil, Kurdistan Region (Iraq)	-	Braidwood,R.J.,Howe,B.PrehistoricInvestigationsinIraqiKurdistan.OrientalInstitute, UniversityofChicago,StudiesinAncientOrientalCivilization,**31** (1960).
4	Cheshmaehsangi 4	33.92	46.71	Kermanshah	6	Heydari-Guran, S. & Ghasidian, E. Late Pleistocene hominin settlement patterns and population dynamics in the Zagros Mountains: Kermanshah region. *Archaeological Research in Asia* **21**, 100161 (2020).
5	Chehelmard 5	34.12	45.94	Kermanshah	25	Heydari-Guran, S. & Ghasidian, E. Late Pleistocene hominin settlement patterns and population dynamics in the Zagros Mountains: Kermanshah region. *Archaeological Research in Asia* 21, 100161 (2020).
6	Ashkaft-e Cham-e Emam Hasan	34.37	45.74	Kermanshah	11	Heydari-Guran, S. & Ghasidian, E. Late Pleistocene hominin settlement patterns and population dynamics in the Zagros Mountains: Kermanshah region. *Archaeological Research in Asia* 21, 100161 (2020).
7	Warwasi	34.38	47.16	Kermanshah	0	Dibble, H.L. & Holdaway, S.J. The Middle Paleolithic industries of Warwasi. In D.I. Olszewski and H. L. Dibble eds. The Paleolithic prehistory of the Zagros-Taurus. The University Museum, Pennsylvania, pp. 75-100 (1993).
8	Bisetun	34.39	47.43	Kermanshah	Neanderthal	Coon, C.S. The Seven Caves. New York: Knopf (1957).
9	Do-Ashkaft	34.39	47.12	Kermanshah	-	Biglari, F. & Heydari, S. Do-Ashkaft: A recent discovery Mousterian cave site in Kermanshah Plain, Iran. *Antiquity* **75**, 487-488 (2001).

10	Kobeh	34.43	47.17	Kermanshah	0	Marean, C. W. & Kim, S. Y. Mousterian large mammal remains from Kobeh Cave: behavioral implications for Neanderthals and early modern humans. *Current Anthropology* **39**, 79-113 (1998).
11	Ashkaft-e Dariwa	34.46	46.17	Kermanshah	6	Heydari-Guran, S. & Ghasidian, E. Late Pleistocene hominin settlement patterns and population dynamics in the Zagros Mountains: Kermanshah region. *Archaeological Research in Asia* 21, 100161 (2020).
12	Ashkaft-e Baluch cave	34.58	46.97	Kermanshah	30	Heydari-Guran, S. & Ghasidian, E. Late Pleistocene hominin settlement patterns and population dynamics in the Zagros Mountains: Kermanshah region. *Archaeological Research in Asia* 21, 100161 (2020).
13	Vezmeh	34.05	46.64	Kermanshah	Neanderthal	Heydari-Guran, S. & Ghasidian, E. Late Pleistocene hominin settlement patterns and population dynamics in the Zagros Mountains: Kermanshah region. *Archaeological Research in Asia* 21, 100161 (2020).
14	Bawa Yawan	34.63	46.93	Kermanshah	Neanderthal	HeydariGuran,S.,Ghasidian,E.,TheMUPZagrosProject:trackingtheMiddleUpperPalaeolithictransitionintheKermanshahregion,west-centralZagros. Iran. *Antiquity* **91**, 355 (2017).
15	Hazar Merd	35.49	45.31	Kurdistan	-	Garrod, D.A.E. The Palaeolithic of Southern Kurdistan: Excavations in the Caves of Zarzi and Hazar Merd. *Bulletin of the American school of Prehistoric Research* **6**, 9-43 (1930).
16	Mar Tarik Cave	34.39	47.43	Kermanshah	-	Jaubert, J. et al. New Research on Paleolithic of Iran: Preliminary Report of 2004 Iranian-French Joint Mission. Archaeological Reports 4. Iranian Center for Archaeological Research, Tehran. 17-26 (2006).
17	Amar Merdeg	33.17	46.24	Ilam	0	Biglari, F., G. Nokandeh, and S. Heydari. A recent find of a Paleolithic assemblage from the foothills of the Zagros Mountains. *Antiquity* **74**, 749–50 (2000).
18	Kunji	33.44	48.35	Lurestan	3	Hole, F., and Flannery, K.V. The prehistory of southwestern Iran: A preliminary report. *Proceedings of the Prehistoric Society* **33**, 147–206 (1967).<br>Baumler, M. and Speth, J. D. A Middle Paleolithic assemblage from Kunji Cave, Iran. In D.I. Olszewski and H.L. Dibble eds. The Paleolithic prehistory of the Zagros-Taurus. The University Museum, Pennsylvania, 1-74 (1993).
19	Ghmari	33.50	48.33	Lurestan	1	Hole, F., and Flannery, K.V. The prehistory of southwestern Iran: A preliminary report. *Proceedings of the Prehistoric Society* **33**, 147–206 (1967).
20	Gachi	33.50	48.22	Lurestan	3	Roustaei, K., Vahdati Nasab, H., Biglari, F., Heydari, S., Clark, G.A., and Lindly, J.M. 2004. Recent Paleolithic surveys in Lurestan. *Current Anthropology* **45**, 692–707.
21	Bard Spid	33.64	47.60	Lurestan	-	MacBurney, C.B.M. Paleolithic excavations in the Zagros mountains. Iran VIII, 185-6 (1970).
22	Qaleh Bozi 2	32.41	51.55	Isfahan	57	Biglari, F. et al. Test excavation at the Middle Paleolithic sites of Qaleh Bozi, southwest of Central Iran, A preliminary report. In M. Otte, F. Biglari and J. Jaubert, eds. Iran Paleolithic/ Le Paléolithique d'Iran. BAR International series, 29-38 (2009).

23	Hol Abad I	33.58	52.00	Isfahan	36	Heydari-Guran, S. Palaeolithic Landscapes of Iran. BAR International Series, 2568 (2014).
24	QG8	33.66	52.11	Isfahan	71	Heydari-Guran, S. Palaeolithic Landscapes of Iran. BAR International Series, 2568 (2014).
25	QG9	33.67	52.18	Isfahan	14	Heydari-Guran, S. Palaeolithic Landscapes of Iran. BAR International Series, 2568 (2014).
26	Kaftarkhun	33.90	51.37	Isfahan	0	Heydari-Guran, S. Palaeolithic Landscapes of Iran. BAR International Series, 2568 (2014).
27	Niasar	33.96	51.14	Isfahan	0	Heydari-Guran, S. Palaeolithic Landscapes of Iran. BAR International Series, 2568 (2014).
28	ZA 16	35.37	50.55	Markazi	-	Heydari-Guran, S. Palaeolithic Landscapes of Iran. BAR International Series, 2568 (2014).
29	Mirak	35.54	53.47	Semnan	-	Vahdati Nasab, H. Clark, G.A., Torkmandi, S. Three Major Late Pleistocene Dispersal Corridors across the Iranian Plateau: A Case Study from Mirak, a Middle Paleolithic Site at the Northern Edge of the Iranian Central Desert (Dash-e-Kavir). Quaternary International, **300**: 267-281 (2013).
30	Chal Tapeh	36.59	47.24	Azarbiyjan Gharbi	58	Heydari-Guran, S., Ghasidian, E. and Conard, N. J. 2009. Paleolithic Sites on Travertine and Tufa Formations in Iran. In M. Otte, F. Biglari and J. Jaubert eds. Iran Paleolithic / Le Paléolithique d'Iran. BAR International series, 109-124 (2009).<br>Heydari-Guran, S. Palaeolithic Landscapes of Iran. BAR International Series, 2568 (2014).
31	Tamtameh	37.49	44.74	Azarbaijan Gharbi	4	Coon, C.S. The Seven Caves. New York: Knopf (1957).
32	Kaldar Cave	33.55	48.29	Lurestan	-	Bazgir, B. et al. Understanding the emergence of modern humans and the disappearance of Neanderthals: Insights from Kaldar Cave (Khorramabad Valley, Western Iran). Sci. Rep.**7**, 43460 (2017).
33	Houmian Rockshelter	33.64	47.60	Lurestan	-	MacBurney, C.B.M. Paleolithic excavations in the Zagros mountains. Iran VIII, 185-6 (1970).
34	Ghar Huchi	33.75	47.09	Hulalian	-	Mortensen, P. Paleolithic and Epipaleolithic sites in the Hulailan Valley, Northern Luristan. In D. Olszewski and H. Dibble, eds. The Paleolithic Prehistory of the Zagros-Tauros. The University Museum of Archaeology and Anthropology, University of Pennsylvania, Philadelphia, 159186 (1993).
35	Eshkaft-e Gavi	29.87	52.74	Fars	-	Rosenberg, M. Paleolithic settlement pattern in the Marv Dasht, Fars Province, Iran. Unpublished Ph.D. dissertation. University of Pennsylvania. USA (1988).
36	Jahrom	28.55	53.70	Fars	86	Piperno, M. Jahrom, a Middle Paleolithic Site in Fars, Iran. *East and West*, **22**: 183-97 (1972).
37	K9-5	29.79	53.21	Fars	6	Rosenberg, M. Paleolithic settlement pattern in the Marv Dasht, Fars Province, Iran. Unpublished Ph.D. dissertation. University of Pennsylvania. USA (1988).

38	LPC	29.80	52.63	Fars	0	Rosenberg, M. Paleolithic settlement pattern in the Marv Dasht, Fars Province, Iran. Unpublished Ph.D. dissertation. University of Pennsylvania. USA (1988).
39	KZC	29.94	52.64	Fars	0	Rosenberg, M. Paleolithic settlement pattern in the Marv Dasht, Fars Province, Iran. Unpublished Ph.D. dissertation. University of Pennsylvania. USA (1988).
40	BZC	30.08	52.73	Fars	11	Rosenberg, M. Paleolithic settlement pattern in the Marv Dasht, Fars Province, Iran. Unpublished Ph.D. dissertation. University of Pennsylvania. USA (1988).
41	BAC	30.09	52.41	Fars	7	Rosenberg, M. Paleolithic settlement pattern in the Marv Dasht, Fars Province, Iran. Unpublished Ph.D. dissertation. University of Pennsylvania. USA (1988).
42	BBC	30.09	52.41	Fars	4	Rosenberg, M. Paleolithic settlement pattern in the Marv Dasht, Fars Province, Iran. Unpublished Ph.D. dissertation. University of Pennsylvania. USA (1988).
43	Borz	30.28	51.45	Fars	95	Heydari-Guran, S. Palaeolithic Landscapes of Iran. BAR International Series, 2568 (2014).
44	Shiv	30.38	51.31	Fars	2	Heydari-Guran, S. Palaeolithic Landscapes of Iran. BAR International Series, 2568 (2014).
45	Khanahmad II	30.30	51.09	Kohgiluyeh and Boyer-Ahmad	0	Heydari-Guran, S. Palaeolithic Landscapes of Iran. BAR International Series, 2568 (2014).
